# FGF-23 correlates with endocrine and metabolism dysregulation, worse cardiac and renal function, inflammation level, stenosis degree, and independently predicts in-stent restenosis risk in coronary heart disease patients underwent drug-eluting-stent PCI

**DOI:** 10.1186/s12872-020-01839-w

**Published:** 2021-01-07

**Authors:** Tingting Song, Yang Fu, Yanbo Wang, Wei Li, Jiayu Zhao, Xun Wang, Haiyan Wang, Ying Zhao, Xianghua Fu

**Affiliations:** Department of Cardiology, The Second Hospital of Hebei Medical University, 215 West Heping Road, Shijiazhuang, 050000 China

**Keywords:** Fibroblast growth factor 23, In-stent restenosis, Drug-eluting stent, Coronary heart disease, Risk factors

## Abstract

**Background:**

The present study aimed to assess the correlation of fibroblast growth factor (FGF)-23 expression with clinical characteristics, then further explore its value in predicting 2-year in-stent restenosis (ISR) risk in coronary heart disease (CHD) patients underwent percutaneous coronary intervention (PCI) with drug-eluting stent (DES).

**Methods:**

In this prospective, single-center, observational study, totally 214 CHD patients treated by PCI with DES were consecutively recruited, and peripheral blood samples were collected prior to PCI with DES for serum samples isolation. Following, FGF-23 level in the serum samples was detected via enzyme linked-immuno-sorbent Assay. The follow-up coronary angiography was performed at 1 year and 2 years after PCI or if suspected ISR symptoms occurred.

**Results:**

FGF-23 was positively correlated with fasting blood-glucose, insulin resistance, serum creatinine, serum uric acid, LDL-C, high-sensitivity C-reactive protein, cardiac troponin I and N-terminal-proB-type natriuretic peptide, while was negatively associated with HDL-C and left ventricular ejection fraction (all *P* < 0.01). Furthermore, FGF-23 was positively correlated with hypercholesteremia, hyperuricemia and family history of CAD (all *P* < 0.05). However, it did not correlate with other chronic complications, biochemical indexes, lesion features or PCI parameters (all *P* > 0.05). Moreover, FGF-23 level was higher in 2-year ISR patients (n = 38) compared to 2-year non-ISR patients (n = 176) (*P* < 0.001), and receiver operating characteristic curve indicated that FGF-23 was of good value in predicting 2-year ISR risk (AUC 0.828, 95% CI 0.761–0.896).

**Conclusion:**

FGF-23 correlates with endocrine and metabolism dysregulation, worse cardiac and renal function, inflammation level, stenosis degree of target lesion, and serves as an independent risk factor for 2-year ISR risk in CHD patients underwent PCI with DES.

## Background

Coronary heart disease (CHD) remains to be the major contributor of mortality and is responsible for one third of all deaths worldwide [1, 2]. Recently, despite the standard treatment of coronary artery bypass grafting (CABG) in CHD patients, percutaneous coronary intervention (PCI) with drug-eluting stents (DES) implantation has been recommended as a valid alternative routine treatment approach for revascularization in increasing proportion of CHD patients [3, 4]. Although PCI with DES implantation effectively alleviates stenosis of targeted coronary artery lesions and lowers the incidence of major adverse cardiac and cerebrovascular events (MACE), in-stent restenosis (ISR) continues to be a serious complication with the incidence of 5%-20% within 5 years [5, 6]. Therefore, it is essential to discover novel biomarkers for predicting ISR risk to sustain long-term efficacy in CHD patients underwent PCI with DES.

Fibroblast growth factor (FGF)-23, a member of FGF family, is an endocrine hormone correlates with calcium-phosphate homoeostasis, which is primarily expressed and secreted by osteocytes [7]. Initially, FGF-23 is reported to be implicated in the regulation of phosphatemia and mineral metabolism via interacting with its co-receptor klotho in kidney, and growing recent clinical evidences demonstrate the regulatory role of FGF-23 in diverse pathological processes of cardiovascular diseases (such as: coronary artery stenosis, myocardial fibrosis, cardiac injury, atherosclerosis, vascular calcification) and potential of FGF-23 as a clinical biomarker for predicting higher risk of adverse cardiovascular outcomes [8–10]. For example, FGF-23 is associated with accelerated plaque calcification as well as advanced severity of atherosclerosis in terms of vascular plaque burden, and it functions as a risk factor for coronary artery stenosis as well as adverse cardiovascular outcomes in CHD patients [11]. In addition, one clinical study reveals that FGF-23 is associated with carotid artery intima-media thickness, left ventricular mass, unfavorable cardiac function, and serves as an indicator for atherosclerosis in patients with gestational diabetes [12]. The aforementioned data suggests the contribution of FGF-23 on development of cardiovascular injury and coronary stenosis, therefore, we hypothesized that FGF-23 might be also correlated with ISR risk in CHD patients treated by PCI with DES, however, study related to the predictive potential of FGF-23 in ISR risk has not been conducted yet. The present study was conducted to detect the correlation of FGF-23 expression with clinical characteristics, and further to explore its value in predicting 2-year ISR risk in CHD patients underwent PCI with DES.

## Methods

### Study population

In this prospective, single-center, observational study, 214 CHD patients treated by PCI with DES in The Second Hospital of Hebei Medical University between January 2016 and June 2018 were consecutively recruited. The inclusion criteria were: (1) diagnosed to be CHD; (2) age ≥ 45 years old; (3) suitable for PCI with DES; (4) willing to provide blood samples for study; (5) able to be regularly followed up. The exclusion criteria included: (1) history of PCI, coronary artery bypass grafting, or other cardiovascular surgery; (2) history of heart transplantation before study; (3) complicated with other heart diseases; (4) active infection or inflammation; (5) severe dysfunction in liver or kidney; (6) history of hematological system diseases or malignancies; (7) pregnant or lactating female patients. This study was approved by the Ethic Committee of The Second Hospital of Hebei Medical University, and all patients signed the informed consents.

### Clinical data collection

Patients’ age, gender and body mass index (BMI) were collected from basic inquiring and examination. The current smoke status, hypertension, diabetes mellitus, hypercholesteremia, hyperuricemia, and family history of coronary artery disease (CAD) were collected by history taking. Mean arterial pressure (MAP) was calculated after measurement of blood pressure. Left ventricular ejection fraction (LVEF) was evaluated by ultrasonic cardiogram. Following indexes were documented after general examination and biochemical test: biochemical index (fasting blood-glucose (FBG), insulin resistance index (IRI) [13], glycated hemoglobin, triglyceride (TG), total cholesterol (TC), low-density lipoprotein cholesterol (LDL-C), high-density lipoprotein cholesterol (HDL-C), high-sensitivity C-reactive protein (Hs-CRP), erythrocyte sedimentation rate (ESR), white blood cell (WBC), neutrophil, serum creatinine (Scr) and serum uric acid (SUA), cardiac troponin I (cTnl) and N-terminal probrain natriuretic peptide (NT-proBNP). Lesion features were obtained by image examination, which included multivessel artery lesions, location of target lesion, two target lesions, stenosis degree of target lesion and length of target lesion. In addition, PCI parameters including length of stent, diameter of stent, time of stent dilation and balloon dilation pre stent, and drug management (aspirin, nitrates, statins, β receptor blockers, angiotensin converting enzymes inhibitors (ACEIs), angiotensin receptor blockers (ARBs) and calcium channel blockers) were documented after operation. In addition, among patients with type 2 diabetes mellitus, the incidence of non-ST-segment elevation myocardial infarction (NSTEMI) and ST-segment elevation myocardial infarction (STEMI) were also documented.

### Sampling and quantitative determination

Peripheral blood samples of enrolled patients were collected using promoting coagulating tubes before PCI with DES. After collecting, the samples were placed at room temperature to clot for 15 min, then serum samples were isolated by centrifuging at 1000×*g* for 15 min in a refrigerated centrifuge. The FGF-23 level in the serum samples was detected using Human FGF-23 Enzyme Linked-Immuno-Sorbent Assay (ELISA) Kit (Invitrogen, Carlsbad, California, USA). The Human FGF-23 ELISA kit presented with analytical sensitivity of 300 pg/mL with an assay range of 300–7500 pg/mL. The sample preparation, standards diluting and ELISA performing were carried out in accordance with the kit instructions (available on: https://assets.thermofisher.com/TFS-Assets/LSG/manuals/EH189RB.pdf). The absorbance of each plate was obtained at 450 nm within 30 min after adding the Stop Solution. Standard curve was generated using curve-fitting software, and concentration for unknown samples and control was read from the standard curve.

### Follow-up and ISR assessment

The PCI procedures, DES implantation and postoperative management were performed according to PCI guideline [14]. During hospitalization, coronary angiography was conducted before PCI and immediately post PCI. After discharge, follow-up coronary angiography was performed at 1 year after PCI and 2 years after PCI. Meanwhile, coronary angiography was also performed if suspected ISR symptoms occurred during follow-up. The ISR was assessed by quantitative coronary angiography (QCA), and the procedures of QCA were carried out according to previous studies [15, 16]. In brief, after administration of intracoronary nitroglycerin (100 to 300 µg), standard angiographic images were obtained so that each coronary segment was recorded in at least 2 orthogonal views. Images were recorded either on a DICOM-formatted CD or on cine film. For restenosis assessment, a projected diameter was defined by projecting the object with a parallel light source and calculating the length of the shadow on a plane, which was perpendicular to the light source. The minimum and maximum projected diameter (D_min_ and D_max_) were found by rotating the object around the center of gravity three times at a step of 30°. For each rotation, the projection distances on the X- and Y-axis were calculated and compared to the previous minimum and maximum. Diameter stenosis was calculated as: (1 − D_min_/D_max_) × 100% in each cross-section, and the maximum value was taken for restenosis assessment. The 2-year ISR was defined as the lumen stenosis of stent-implanted segment at 2-year follow-up exceeded 50% compared with lumen assessed immediately after PCI [17]. Patients who lost follow-up before ISR occurrence had been excluded from study. All 214 patients were divided into 2-year ISR patients and 2-year non-ISR patients according to whether the ISR occurred during the follow-up.

### Statistical analysis

All data analyses were carried out using SPSS 24.0 statistical software (SPSS Inc, Chicago, Illinois, USA), and all graphs were plotted using GraphPad Prism 8.01 software (GraphPad Software Inc, San Diego, USA). Kolmogorov–Smirnov test was carried out to determine the normality of continuous data. Normal-distributed continuous data were displayed as mean with standard deviation (SD), and Skewed-distributed continuous data were exhibited to be median with interquartile range (IQR). The categorical data were shown to be number with percentage (No. (%)). Student’s *t* test or Wilcoxon rank sum test was used to determine the continuous data between two groups. Chi-square test was used to determine the categorical data between two groups. Correlation was analyzed by Spearman rank correlation test. Receiver operating characteristic (ROC) curve analysis was plotted, and the area under the curve (AUC) with 95% confidence interval (CI) was used to identify optimal cutoff values of FGF-23 with maximum sensitivity and specificity for prediction of 2-year ISR. Factors with *P* value < 0.05 in the univariate logistic regression were further included in the multivariate logistic regression analysis [forward stepwise (conditional)] to generate the 2-year ISR risk prediction model, and the predictive performance of the model was further assessed by ROC curve. *P* value < 0.05 was considered to be significant.

## Results

### Clinical baseline characteristics of CHD patient

The mean age of CHD patients was 64.6 ± 9.1 years, and there were 40 (18.7%) females and 174 (81.3%) males. The detailed baseline information of BMI, current smoke, chronic complications, biochemical indexes, lesion features, PCI parameters, and post-surgery medications among all CHD patients were displayed in Table 1. In addition, all patients were classified as 2-year ISR patients (n = 38) and 2-year non-ISR patients (n = 176) according to whether the ISR occurred during the study follow-up. No difference of age, gender, BMI, current smoke, hypertension, family history of CAD, MAP, SUA, TG, TC, ESR, WBC, neutrophil, LVEF, cTnI, target lesion at LAD, target lesion at RCA, stenosis degree of target lesion, diameter of stent, time of stent dilation, pre-stent balloon dilation, β-receptor blockers or calcium channel blockers was observed between 2-year non-ISR patients and 2-year ISR patients (all *P* > 0.05). However, the proportion of patients with diabetes mellitus, hypercholesteremia, hyperuricemia, multivessel artery lesions, two target lesions, target lesion at LCX, ACEIs/ARBs, and the level of FBG, IRI, Scr, LDL-C, HsCRP, NT-proBNP, length of target lesion, length of stent were increased, while the level of HDL-C was decreased in 2-year ISR patients compared with 2-year non-ISR patients (all *P* < 0.05) (Table 1).

**Table 1:** Characteristics of CHD patients

Items	Total patients (N = 214)	2-year non-ISR patients (n = 176)	2-year ISR patients (n = 38)	*P* value
Age (years), mean ± SD	64.6 ± 9.1	64.2 ± 8.9	66.3 ± 10.0	0.199
Gender, No. (%)				0.155
Female	40 (18.7)	36 (20.5)	4 (10.5)	
Male	174 (81.3)	140 (79.5)	34 (89.5)	
BMI (kg/m^2^), mean ± SD	24.5 ± 3.6	24.5 ± 3.7	24.9 ± 3.1	0.462
Current smoke, No. (%)	55 (25.7)	43 (24.4)	12 (31.6)	0.361
Hypertension, No. (%)	150 (70.1)	119 (67.6)	31 (81.6)	0.088
Diabetes mellitus, No. (%)	63 (29.4)	43 (24.4)	20 (52.6)	**0.001**
Hypercholesteremia, No. (%)	135 (63.1)	104 (59.1)	31 (81.6)	**0.009**
Hyperuricemia, No. (%)	73 (34.1)	53 (30.1)	20 (52.6)	**0.008**
Family history of CAD, No. (%)	46 (21.5)	34 (19.3)	12 (31.6)	0.095
MAP (mmHg), median (IQR)	106.0 (97.0–115.0)	105.0 (96.3–116.8)	107.0 (102.0–111.3)	0.350
FBG (mmol/L), mean ± SD	5.8 ± 1.2	5.7 ± 1.3	6.2 ± 0.9	**0.019**
IRI, mean ± SD	2.25 ± 1.6	2.1 ± 1.5	3.0 ± 1.5	**0.001**
Scr (μmol/L), mean ± SD	82.7 ± 15.6	81.5 ± 14.8	88.1 ± 18.2	**0.017**
SUA (μmol/L), median (IQR)	333.6 (285.9–408.2)	333.3 (289.1–400.0)	365.3 (279.1–447.5)	0.309
TG (mmol/L), median (IQR)	1.8 (1.0–2.4)	1.8 (1.0–2.4)	1.9 (1.0–2.8)	0.587
TC (mmol/L), median (IQR)	4.6 (3.9–5.2)	4.5 (3.8–5.0)	4.6 (4.3–5.6)	0.069
LDL-C (mmol/L), mean ± SD	2.8 ± 0.6	2.7 ± 0.6	3.1 ± 0.6	**0.002**
HDL-C (mmol/L), mean ± SD	1.0 ± 0.2	1.0 ± 0.3	0.9 ± 0.2	**0.024**
HsCRP (mg/L), median (IQR)	4.7 (1.8–8.0)	3.9 (1.0–7.1)	7.4 (5.8–10.8)	** < 0.001**
ESR (mm/L), median (IQR)	12.7 (6.7–20.8)	12.3 (5.8–20.7)	13.8 (8.3–26.1)	0.137
WBC (*10^9^/L), median (IQR)	6.1 (4.9–7.1)	6.1 (4.8–7.1)	6.3 (5.1–7.3)	0.367
Neutrophil (*10^9^/L), mean ± SD	3.4 ± 1.0	3.4 ± 1.0	3.6 ± 1.1	0.304
LVEF (%), median (IQR)	64.0 (59.8–70.0)	64.0 (60.0–70.0)	64.0 (57.8–70.0)	0.539
cTnI (pg/mL), median (IQR)	29.5 (17.8–47.7)	29.4 (15.3–46.9)	35.5 (23.5–57.2)	0.053
NT-proBNP (pg/mL), median (IQR)	76.5 (46.8–126.1)	75.6 (44.8–122.8)	78.1 (67.0–171.6)	**0.039**
Multivessel artery lesions, No. (%)	164 (76.6)	127 (72.2)	37 (97.4)	**0.001**
Patients with two target lesions, No. (%)	77 (36.0)	56 (31.8)	21 (55.3)	**0.006**
Target lesion at LAD, No. (%)	128 (59.8)	104 (59.1)	24 (63.2)	0.643
Target lesion at LCX, No. (%)	82 (38.3)	61 (34.7)	21 (55.3)	**0.018**
Target lesion at RCA, No. (%)	81 (37.9)	67 (38.1)	14 (36.8)	0.888
Stenosis degree of target lesion (%), median (IQR)	86.0 (82.0–89.0)	86.0 (82.0–89.0)	86.0 (83.0–91.0)	0.111
Length of target lesion (mm), median (IQR)	33.5 (26.0–40.0)	33.0 (26.0–39.0)	37.0 (29.0–46.3)	**0.018**
Length of stent (mm), median (IQR)	37.0 (30.0–43.3)	37.0 (28.3–43.0)	40.5 (33.0–49.0)	**0.016**
Diameter of stent (mm), median (IQR)	3.2 (2.9–3.4)	3.3 (2.9–3.4)	3.1 (3.0–3.3)	0.209
Time of stent dilation (s), median (IQR)	16.0 (13.0–18.0)	15.0 (14.0–18.0)	16.5 (12.0–20.0)	0.731
Pre-stent balloon dilation, No. (%)	66 (30.8)	56 (31.8)	10 (26.3)	0.505
Aspirin, No. (%)	214 (100.0)	176 (100.0)	38 (100.0)	–
Nitrates, No. (%)	214 (100.0)	176 (100.0)	38 (100.0)	–
Statins, No. (%)	214 (100.0)	176 (100.0)	38 (100.0)	–
β-receptor blockers, No. (%)	182 (85.0)	152 (86.4)	30 (78.9)	0.245
ACEIs/ARBs, No. (%)	134 (62.6)	116 (65.9)	18 (47.4)	**0.032**
Calcium channel blockers, No. (%)	68 (31.8)	58 (33.0)	10 (26.3)	0.425

Boldface represented *P* < 0.05

*CHD* coronary heart disease, *ISR* in-stent restenosis, *SD* standard deviation, *BMI* body mass index, *CAD* coronary artery disease, *MAP* mean arterial pressure, *IQR* interquartile range, *FBG* fasting blood-glucose, *IRI* insulin resistance index, *Scr* serum creatinine, *SUA* serum uric acid, *TG* triglyceride, *TC* total cholesterol, *LDL-C* low density lipoprotein cholesterol, *HDL-C* high density lipoprotein cholesterol, *HsCRP* high-sensitivity C-reactive protein, *ESR* erythrocyte sedimentation rate, *WBC* white blood cell, *LVEF* left ventricular ejection fraction, *cTnI* cardiac troponin I, *NT-proBNP* N-terminal-proB-type natriuretic peptide, *LAD* left anterior descending branch, *LCX* left circumflex artery, *RCA* right coronary artery, *ACEIs* angiotensin converting enzymes inhibitors, *ARBs* angiotensin receptor blockers

### Correlation of FGF-23 with continuous characteristics in CHD patients

FGF-23 was positively correlated with FBG (r = 0.245, *P* < 0.001), IRI (r = 0.294, *P* < 0.001), Scr (r = 0.381, *P* < 0.001), SUA (r = 0.287, *P* < 0.001), LDL-C (r = 0.187, *P* = 0.006), HsCRP (r = 0.377, *P* < 0.001), cTnl (r = 0.436, *P* < 0.001), NT-proBNP (r = 0.400, *P* < 0.001) and stenosis degree of target lesion (r = 0.182, *P* = 0.008) (Table 2). However, FGF-23 was negatively associated with HDL-C (r = -0.179, *P* = 0.009) and LVEF (r = − 0.272, *P* < 0.001). In addition, no correlation was observed between FGF-23 and age, BMI, MAP, TG, TC, ESR, WBC, neutrophil or length of target lesion in CHD patients (all *P* > 0.05).

**Table 2:** Correlation of FGF-23 with continuous characteristics

Items	FGF-23	
	*P* value	Spearman r
Age	0.199	0.088
BMI	0.113	0.109
MAP	0.378	0.061
FBG	**< 0.001**	0.245
IRI	**< 0.001**	0.294
Scr	**< 0.001**	0.381
SUA	**< 0.001**	0.287
TG	0.093	0.115
TC	0.535	0.043
LDL-C	**0.006**	0.187
HDL-C	**0.009**	− 0.179
HsCRP	**< 0.001**	0.377
ESR	0.448	0.052
WBC	0.455	− 0.051
Neutrophil	0.943	− 0.005
LVEF	**< 0.001**	− 0.272
cTnI	**< 0.001**	0.436
NT-proBNP	**< 0.001**	0.400
Stenosis degree of target lesion	**0.008**	0.182
Length of target lesion	0.321	0.068

Boldface represented *P* < 0.05

*FGF-23* fibroblast growth factor 23, *BMI* body mass index, *MAP* mean arterial pressure, *FBG* fasting blood-glucose, *IRI* insulin resistance index, *Scr* serum creatinine, *SUA* serum uric acid, *TG* triglyceride, *TC* total cholesterol, *LDL-C* low density lipoprotein cholesterol, *HDL-C* high density lipoprotein cholesterol, *HsCRP* high-sensitivity C-reactive protein, *ESR* erythrocyte sedimentation rate, *WBC* white blood cell, *LVEF* left ventricular ejection fraction, *cTnI* cardiac troponin I, *NT-proBNP* N-terminal-proB-type natriuretic peptide

### Correlation of FGF-23 with categorical characteristics in CHD patients

FGF-23 was positively correlated with the occurrence of hypercholesteremia (*P* < 0.001), hyperuricemia (*P* = 0.022) and family history of CHD (*P* = 0.022) (Table 3). However, there was no correlation of FGF-23 with gender, current smoke, hypertension, diabetes mellitus, multivessel artery lesions, two target lesions, target lesion at LAD, target lesion at LCX, or target lesion at RCA in CHD patients.

**Table 3:** Correlation of FGF-23 with categorical characteristics

Items	FGF-23 (pg/mL), median (IQR)	*P* value
Gender		0.946
No	736.6 (634.3–808.5)	
Yes	736.5 (608.0–905.8)	
Current smoke		0.700
No	727.9 (608.2–907.5)	
Yes	752.0 (661.8–855.1)	
Hypertension		0.167
No	706.2 (571.8–887.1)	
Yes	748.1 (641.0–886.0)	
Diabetes mellitus		0.201
No	720.9 (587.0–862.8)	
Yes	749.6 (657.8–907.5)	
Hypercholesteremia		**< 0.001**
No	582.7 (482.5–694.4)	
Yes	798.6 (720.0–988.4)	
Hyperuricemia		**0.022**
No	715.8 (575.8–901.7)	
Yes	766.4 (672.5–870.8)	
Family history of CAD		**0.022**
No	729.5 (603.5–848.3)	
Yes	781.4 (667.5–1005.0)	
Multivessel artery lesions		0.061
No	691.2 (579.7–795.2)	
Yes	750.2 (630.4–914.6)	
Patients with two target lesions		0.880
No	746.5 (630.4–876.7)	
Yes	721.2 (581.9–892.1)	
Target lesion at LAD		0.628
No	720.3 (622.4–909.9)	
Yes	748.9 (603.7–876.9)	
Target lesion at LCX		0.496
No	741.1 (609.5–896.5)	
Yes	734.3 (616.5–886.0)	
Target lesion at RCA		0.306
No	749.6 (636.5–863.3)	
Yes	698.1 (592.8–912.3)	

Boldface represented *P* < 0.05

*FGF-23* fibroblast growth factor 23, *IQR* interquartile range, *CAD* coronary artery disease, *LAD* left anterior descending branch, *LCX* left circumflex artery, *RCA* right coronary artery

### Correlation of FGF-23 with 2-year ISR risk in CHD patients

**Fig. 1:**
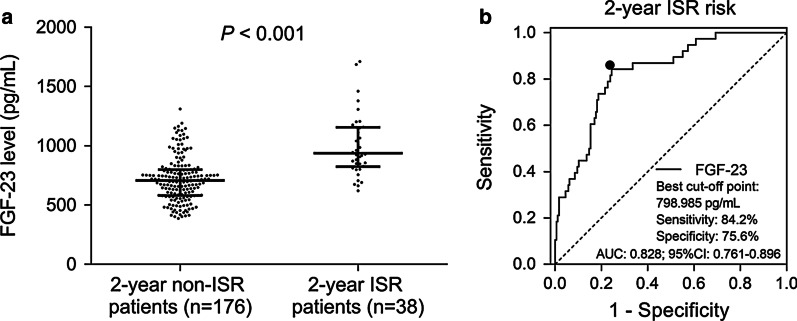
Predictive value of FGF-23 for 2-year ISR risk. Comparison of FGF-23 expression between 2-year non-ISR patients and 2-year ISR patients (**a**). Ability of FGF-23 in predicting 2-year ISR risk in CHD patients treated by PCI with DES (**b**). *FGF-23* fibroblast growth factor 23, *ISR* in-stent restenosis, *PCI* percutaneous coronary intervention, *DES* drug-eluting stent, *CHD* coronary heart disease, *AUC* area under the curve, *CI* confidence interval

FGF-23 level was higher in 2-year ISR patients [936.8 pg/ml (823.6–1155.6 pg/ml)] compared to 2-year non-ISR patients [707.4 pg/ml (581.4–798.5 pg/ml)] (*P* < 0.001) (Fig. 1a). Furthermore, ROC curve indicated that FGF-23 was of good value in predicting 2-year ISR risk (AUC 0.828, 95% CI 0.761–0.896), and the sensitivity as well as specificity at the best cut-off point (798.985 pg/ml) (the point where the largest sum of sensitivity and specificity occurred) were 84.2% and 75.6%, respectively (Fig. 1b).

### Factors affecting 2-year ISR in CHD patients

Univariate logistic regression analysis indicated that FGF-23 (OR = 1.006, *P* < 0.001), diabetes mellitus (OR = 3.437, *P* = 0.001), hypercholesteremia (OR = 3.066, *P* = 0.012), hyperuricemia (OR = 2.579, *P* = 0.009), FBG (OR = 1.429, *P* = 0.020), IRI (OR = 1.391, *P* = 0.002), Scr (OR = 1.027, *P* = 0.020), LDL-C (OR = 2.551, *P* = 0.002), HsCRP (OR = 1.195, *P* < 0.001), multivessel artery lesion (OR = 14.276, *P* = 0.010), two target lesions (OR = 2.647, *P* = 0.008), target lesion at LCX (OR = 2.329, *P* = 0.020), length of target lesion (OR = 1.054, *P* = 0.009), length of stent (OR = 1.053, *P* = 0.010) were positively associated with 2-year ISR risk, however, HDL-C (OR = 0.170, *P* = 0.026) and ACEIs/ARBs (OR = 0.466, *P* = 0.035) were negatively correlated with 2-year ISR risk in CHD patients (Table 4). Further forward stepwise multivariate logistic regression exhibited that FGF-23 (OR = 1.005, *P* = 0.001), diabetes mellitus (OR = 23.654, *P* < 0.001), LDL-C (OR = 4.458, *P* = 0.002), HsCRP (OR = 1.261, *P* < 0.001), two target lesions (OR = 6.305, *P* = 0.002), length of target lesion (OR = 1.128, *P* = 0.001) were independent predictive factors for increased 2-year ISR in CHD patients.

**Table 4:** Factors correlated with 2-year ISR

Items	Logistic regression model			
	*P* value	OR	95% CI	
			Lower	Higher
*Univariate logistic regression*				
FGF-23	**< 0.001**	1.006	1.004	1.008
Age	0.199	1.026	0.987	1.067
Male	0.163	2.186	0.728	6.559
BMI	0.460	1.036	0.943	1.139
Current smoke	0.362	1.428	0.664	3.070
Hypertension	0.094	2.121	0.881	5.108
Diabetes mellitus	**0.001**	3.437	1.667	7.086
Hypercholesteremia	**0.012**	3.066	1.280	7.344
Hyperuricemia	**0.009**	2.579	1.263	5.264
Family history of CAD	0.099	1.928	0.884	4.204
MAP	0.498	1.007	0.987	1.027
FBG	**0.020**	1.429	1.058	1.930
IRI	**0.002**	1.391	1.127	1.716
Scr	**0.020**	1.027	1.004	1.051
SUA	0.241	1.003	0.998	1.007
TG	0.625	1.103	0.745	1.634
TC	0.060	1.426	0.985	2.064
LDL-C	**0.002**	2.551	1.394	4.666
HDL-C	**0.026**	0.170	0.036	0.812
HsCRP	**< 0.001**	1.195	1.099	1.299
ESR	0.180	1.025	0.989	1.063
WBC	0.469	1.085	0.870	1.355
Neutrophil	0.303	1.199	0.848	1.695
LVEF	0.404	0.978	0.927	1.031
cTnI	0.129	1.013	0.996	1.030
NT-proBNP	0.080	1.005	0.999	1.010
Multivessel artery lesion	**0.010**	14.276	1.906	106.910
Patients with two target lesions	**0.008**	2.647	1.296	5.405
Target lesion at LAD	0.643	1.187	0.575	2.449
Target lesion at LCX	**0.020**	2.329	1.144	4.741
Target lesion at RCA	0.888	0.949	0.459	1.961
Stenosis degree of target lesion	0.098	1.071	0.988	1.161
Length of target lesion	**0.009**	1.054	1.013	1.097
Length of stent	**0.010**	1.053	1.012	1.095
Diameter of stent	0.488	0.692	0.244	1.959
Time of stent dilation	0.942	1.003	0.920	1.094
Pre-stent balloon dilation	0.506	0.765	0.348	1.684
β-receptor blockers	0.249	0.592	0.243	1.443
ACEIs/ARBs	**0.035**	0.466	0.229	0.946
Calcium channel blockers	0.427	0.727	0.331	1.597
*Multivariate logistic regression (forward stepwise: conditional)*				
FGF-23	0.001	1.005	1.002	1.007
Diabetes mellitus	< 0.001	23.654	6.374	87.782
LDL-C	0.002	4.458	1.759	11.294
HsCRP	< 0.001	1.261	1.117	1.424
Patients with two target lesions	0.002	6.305	2.015	19.728
Length of target lesion	0.001	1.128	1.054	1.208

Boldface represented *P* < 0.05. Factors with *P* < 0.05 in the univariate logistic regression were further analyzed by multivariate logistic regression with forward stepwise (conditional) method

*ISR* in-stent restenosis, *OR* odds ratio, *CI* confidence interval, *FGF-23* fibroblast growth factor 23, *BMI* body mass index, *CAD* coronary artery disease, *MAP* mean arterial pressure, *FBG* fasting blood-glucose, *IRI* insulin resistance index, *Scr* serum creatinine, *SUA* serum uric acid, *TG* triglyceride, *TC* total cholesterol, *LDL-C* low density lipoprotein cholesterol, *HDL-C* high density lipoprotein cholesterol, *HsCRP* high-sensitivity C-reactive protein, *ESR* erythrocyte sedimentation rate, *WBC* white blood cell, *LVEF* left ventricular ejection fraction, *cTnI* cardiac troponin I, *NT-proBNP* N-terminal-proB-type natriuretic peptide, *LAD* left anterior descending branch, *LCX* left circumflex artery, *RCA* right coronary artery, *ACEIs* angiotensin converting enzymes inhibitors, *ARBs* angiotensin receptor blockers

### Prediction model based on independent risk factors for 2-year ISR risk in CHD patients

**Fig. 2:**
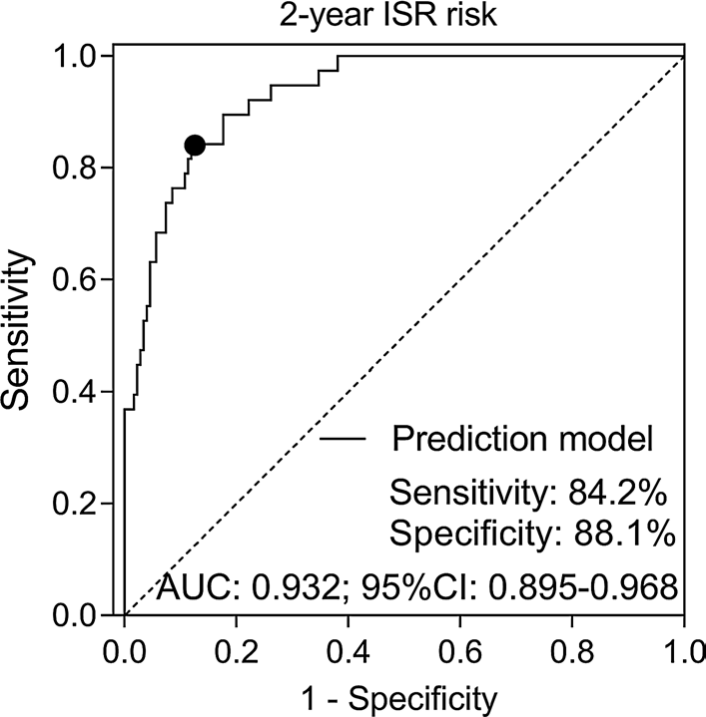
Prediction model involving independent risk factors for 2-year ISR risk. *FGF-23* fibroblast growth factor 23, *ISR* in-stent restenosis, *PCI* percutaneous coronary intervention, *DES* drug-eluting stent, *CHD* coronary heart disease, *AUC* area under the curve, *CI* confidence interval

The predictive performance of the model including independent factors was further assessed, which revealed that the prediction model was of excellent value in predicting 2-year ISR risk (AUC 0.932, 95% CI 0.895–0.968), and the sensitivity as well as specificity at the best cut-off point (the point where the largest sum of sensitivity and specificity occurred) were 84.2% and 88.1%, respectively (Fig. 2).

### Subgroup analysis

Among CHD patients with diabetes mellitus, there were 13 (20.6%) patients with NSTEMI events and 7 (11.1%) patients with STEMI (Additional file 1: Table S1). In patients with NSTEMI events, 9 (69.2%) patients presented with 2-year ISR occurred during the follow-up. As for in patients with STEMI events, 5 (71.4%) patients exhibited 2-year ISR occurred during the follow-up (Additional file 2: Table S2). More information about NSTEMI/STEMI patients with diabetes mellitus was shown in Additional file 2: Table S2.

## Discussion

In the present study, (1) FGF-23 was positively correlated with endocrine and metabolism dysregulation, unfavorable cardiac and renal function, increased inflammation level, and stenosis degree of target lesion in CHD patients underwent PCI with DES. (2) FGF-23 was upregulated in 2-year ISR patients compared with 2-year non-ISR patients, and was of good value in predicting 2-year ISR risk in CHD patients underwent PCI with DES. (3) FGF-23 could function as an independent factor for increased 2-year ISR risk, and the prediction model involving FGF-23 and other risk factors were of excellent value in predicting 2-year ISR risk in CHD patients underwent PCI with DES.

FGF-23 is a bone-derived hormone in response to the increment of circulating phosphate and calcitriol levels, and its implication in the pathogenesis of CKD has been revealed that increased FGF-23 disrupts the bone-kidney-parathyroid hormone balance, which promotes the generation of hyperphosphatemia with the progression of CKD [18]. Recently, several clinical studies suggest that FGF-23, as a pro-inflammatory role, is involved in several aspects of cardiovascular diseases [8, 10, 19, 20]. For example, FGF-23 is reported to be an independent factor for increased stage of peripheral vascular calcified atherosclerotic plaque and carotid artery intima-media thickness, and is responsible for the development and progression of atherosclerosis [21]. In addition, based on the existing evidence, ISR is a complex inflammatory and reparative reaction to vessel injury following stent implantation arising from neointimal hyperplasia, and there is evidence revealing the involvement of regulators which serve as pro-inflammation roles (such as sirtuins, microRNAs (miRNAs)), in the process of atherosclerotic plaque formation, leading to the higher ISR risk [22, 23]. According to these aforementioned evidences, it was speculated that FGF-23 might have value in predicting ISR risk in CHD patients [24]. However, the related evidence is still lack, therefore the present study was performed.

In the present study, 214 CHD patients treated by PCI with DES were enrolled, and the correlation of FGF-23 with the clinical characteristics were further detected in these patients, which observed that FGF-23 was correlated with endocrine and metabolism dysregulation (reflected by indexes including: high level of FBG, IRI, SUA, LDL-C, but low level of HDL-C, presence of hypercholesteremia and hyperuricemia), poor cardiac and renal function (reflected by indexes including: high level of Scr, cTnl and NT-rpoBNP, but low level of LVEF), increased inflammation level (reflected by high level of HsCRP), and stenosis degree of target lesion in CHD patients underwent PCI with DES. The possible reasons for these correlations were listed. (1) Firstly, FGF-23 might enhance the excretion of phosphate per nephron and the secretion of parathyroid hormone (PTH) via dysregulating PTH-calcitriol-phosphate system, thereby contributing to high level of SUA and occurrence of hyperuricemia [8, 25]. (2) Secondly, according to the previous study by Kutluturk etc. [26], FGF-23 was correlated with insulin resistance, impaired glucose tolerance and dysregulation of cholesterol metabolism in obsess population suggesting the correlation of FGF-23 with high FBG level, insulin resistance, abnormal lipoprotein-cholesterol level and presence of hypercholesteremia in CHD patients. (3) Thirdly, FGF-23 was reported to decrease active vitamin D synthesis via decreasing 1α-hydroxylase expression, resulting in low level of anti-inflammation calcitriol in serum, meanwhile, high level of FGF-23 presented enhancing effect on inflammatory mediators and signaling (NF-κB signaling), which both amplifying inflammation [8]. (4) Fourthly, previous study indicated that FGF-23 might trigger adverse cardiovascular remodeling and induce cardiac injury via interplaying with PTH and renin–angiotensin–aldosterone system, thereby leading to high cTnl, NT-proBNP level but low LVEF level [9, 18, 19]. In addition, considering these prominent contributing factors of stenosis including inflammation, cardiac injury and lipid disorder, FGF-23 was therefore correlated with stenosis degree of target lesion in CHD patients underwent PCI with DES. Interestingly, the correlation of FGF-23 with diabetes was not observed, which might be explained by: (1) According to the results in our study, FGF-23 was correlated with increased FBG in CHD patients and higher FBG was considered as a risk factor for pre-diabetes [27], FGF-23 might be correlated with the existence of pre-diabetes via affecting FBG, but not serious enough with diabetes. (2) In addition, the lack of correlation might be attributed to the limited sample size.

Subsequently, followed results indicated that FGF-23 presented good value in predicting 2-year ISR risk, and was an independent predictive factor for increasing 2-year ISR risk in CHD patients underwent PCI with DES. The possible reasons might include that: (1) FGF-23 was positively correlated with endocrine and metabolism dysregulation, poor cardiac and renal function, inflammation level, and stenosis degree of target lesion, which were risk factors for ISR. Therefore, FGF-23 might be correlated with higher risk of ISR via interaction with these factors [3]. (2) Furthermore, given the vascular injury after PCI, FGF-23 might directly induce neointimal pro-hyperplasia and further vascular remodeling via FGF-23/FGF receptor/α-klotho signaling-mediated inflammatory response, which induced the initiation and development of ISR [5, 28]. Therefore, FGF-23 serves as an independent predictive factor for increased risk of ISR in CHD patients underwent PCI with DES. In addition, the results exhibited that diabetes mellitus, LDL-C, HsCRP, two target lesions and length of target lesion were also independent factors for greater 2-year ISR risk in CHD patients underwent PCI with DES, which was in consistence with the previous evidence about the risk factors for restenosis after implantation of DES [3, 5, 29, 30]. The possible mechanisms might be included as follows: (1) Activation of carbonic anhydrases might lead to increased production of reactive oxygen species, further leading to maladaptive cardiac remodelling as well as increased risk of restenosis [31]. (2) Based on previous evidence by Marfella etc. [32], diabetes and CHD both present deregulation of ubiquitin-proteason system, which was regarded as the common pathogenic factor promoting the initial of atherosclerosis and higher risk of ISR [32]. (3) HsCRP-induced activated immune response might affect endothelial function adversely and leading to instability of immunity-dependent atherosclerotic plaque progression [33]. In addition, based on the previous evidence that in both patients with NSTEMI [34] and STEMI [35] events, the close correlation of diabetes mellitus with poor prognosis are observed, and considering the importance of diabetes mellitus in predicting ISR risk in study herein, we detected the ISR risk in NSTEMI/STEMI patients with diabetes mellitus, which found that among CHD patients with diabetes mellitus, there were 69.2% and 71.4% patients with NSTEMI events and patients with STEMI, respectively, who presented with 2-year ISR occurred during the follow-up. Furthermore, the prediction model involving all independent risk factors (including FGF-23) for 2-year ISR improved the predictive value of single indexes, which suggested that FGF-23 could promote accuracy of ISR prediction in CHD patients underwent PCI with DES.

The present study existed some limitations: (1) This was a single-center study with relatively small sample size, thus further studies including more patients from multiple centers were needed for validation. (2) The present study did not explore the implication of FGF-23 in pathophysiologic mechanism of ISR, which needed to be investigated in further experimental researches. (3) The predictive value of FGF-23 for 2-year ISR risk was detected in the current study, while the predictive value of FGF-23 for long-term ISR needed to be further detected. (4) Considering that endothelial dysfunction was reported as an important determinant of development of CHD, further studies that explored the correlation of FGF-23 with endothelial dysfunction were needed [36].

## Conclusion

In conclusion, FGF-23 correlates with endocrine and metabolism dysregulation, worse cardiac and renal function, inflammation level, stenosis degree of target lesion, and serves as an independent risk factor for 2-year ISR risk in CHD patients underwent PCI with DES.

## Supplementary Information

**Additional file 1: Table S1**. NSTEMI/STEMI occurrence within 2 years after PCI in CHD patients with diabetes mellitus.

**Additional file 2: Table S2**. Disease features of NSTEMI/STEMI patients with diabetes mellitus.

## Data Availability

The datasets generated and analyzed during the current study are available from the corresponding author on reasonable request.

## Abbreviations

CHD: Coronary heart disease
CABG: Coronary artery bypass grafting
PCI: Percutaneous coronary intervention
DES: Drug-eluting stents
MACE: Major adverse cardiac and cerebrovascular events
ISR: In-stent restenosis
FGF: Fibroblast growth factor
BMI: Body mass index
CAD: Coronary artery disease
MAP: Mean arterial pressure
LVEF: Left ventricular ejection fraction
FBG: Fasting blood-glucose
IRI: Insulin resistance index
TG: Triglyceride
TC: Total cholesterol
LDL-C: Low-density lipoprotein cholesterol
HDL-C: High-density lipoprotein cholesterol
Hs-CRP: High-sensitivity C-reactive protein
ESR: Erythrocyte sedimentation rate
WBC: White blood cell
Scr: Serum creatinine
SUA: Serum uric acid
cTnl: Cardiac troponin I
NT-proBNP: N-terminal probrain natriuretic peptide
ACEIs: Angiotensin converting enzymes inhibitors
ARBs: Angiotensin receptor blockers
ELISA: Enzyme Linked-Immuno-Sorbent Assay
QCA: Quantitative coronary angiography
SD: Standard deviation
IQR: Interquartile range
ROC: Receiver operating characteristic
AUC: Area under the curve
CI: Confidence interval
