# Supplementary material for: FGF-23 correlates with endocrine and metabolism dysregulation, worse cardiac and renal function, inflammation level, stenosis degree, and independently predicts in-stent restenosis risk in coronary heart disease patients underwent drug-eluting-stent PCI
Source: BMC Cardiovasc Disord. 2021 Jan 7;21:24. doi: 10.1186/s12872-020-01839-w (PMC7791850; doi:10.1186/s12872-020-01839-w)
Supplement: Supplementary file 1 — Additional file 1: Table S1. NSTEMI/STEMI occurrence within 2 years after PCI in CHD patients with diabetes mellitus. [file 12872_2020_1839_MOESM1_ESM.docx]

**Table S1.** NSTEMI/STEMI occurrence within 2 years after PCI in CHD patients with diabetes mellitus

| Items | CHD patients with diabetes mellitus (n=63) |
| --- | --- |
| NSTEMI, No. (%) | 13 (20.6) |
| STEMI, No. (%) | 7 (11.1) |

CHD, coronary heart disease; NSTEMI, non-ST-segment elevation myocardial infarction; STEMI, ST-segment elevation myocardial infarction; PCI, percutaneous coronary intervention.
