# Supplementary material for: FGF-23 correlates with endocrine and metabolism dysregulation, worse cardiac and renal function, inflammation level, stenosis degree, and independently predicts in-stent restenosis risk in coronary heart disease patients underwent drug-eluting-stent PCI
Source: BMC Cardiovasc Disord. 2021 Jan 7;21:24. doi: 10.1186/s12872-020-01839-w (PMC7791850; doi:10.1186/s12872-020-01839-w)
Supplement: Supplementary file 2 — Additional file 2: Table S2. Disease features of NSTEMI/STEMI patients with diabetes mellitus. [file 12872_2020_1839_MOESM2_ESM.docx]

**Table S2.** Disease features of NSTEMI/STEMI patients with diabetes mellitus

| Items | NSTEMI patients (n=13) | STEMI patients (n=7) |
| --- | --- | --- |
| Multivessel artery lesions, No. (%) | 9 (69.2) | 4 (57.1) |
| Two target lesions, No. (%) | 3 (23.1) | 2 (28.6) |
| Target lesion at LAD, No. (%) | 7 (53.8) | 4 (57.1) |
| Target lesion at LCX, No. (%) | 4 (30.8) | 3 (42.9) |
| Target lesion at RCA, No. (%) | 5 (38.5) | 2 (28.6) |
| Stenosis degree of target lesion (%) | 89.0 (87.5-91.0) | 78.0 (76.0-85.0) |
| 2-year ISR, No. (%) | 9 (69.2) | 5 (71.4) |

NSTEMI, non-ST-segment elevation myocardial infarction; STEMI, ST-segment elevation myocardial infarction; LAD, left anterior descending branch; LCX, left circumflex artery; RCA, right coronary artery; ISR, in-stent restenosis.
